# Anti-NMDA Receptor Encephalitis in a Patient with Previous Psychosis and Neurological Abnormalities: A Diagnostic Challenge

**DOI:** 10.1155/2015/253891

**Published:** 2015-06-14

**Authors:** R. David Heekin, Maria C. Catalano, Alfred T. Frontera, Glenn Catalano

**Affiliations:** ^1^University of South Florida Morsani College of Medicine, 12901 Bruce B. Downs Boulevard, Tampa, FL 33612, USA; ^2^James A. Haley Veterans' Hospital, 13000 Bruce B. Downs Boulevard, Tampa, FL 33612, USA; ^3^Department of Neurology, University of South Florida Morsani College of Medicine, 12901 Bruce B. Downs Boulevard, MDC 55, Tampa, FL 33612, USA; ^4^Department of Psychiatry and Behavioral Neurosciences, University of South Florida Morsani College of Medicine, 3515 E. Fletcher Avenue, Tampa, FL 33613, USA

## Abstract

Anti-N-methyl-D-aspartate (NMDA) receptor encephalitis is an autoimmune disorder characterized by IgG autoantibodies directed against the NR1 subunit of the NMDA glutamate receptor. Psychiatric symptoms are common and include psychosis, mania, depressed mood, aggression, and speech abnormalities. Neurological symptoms such as seizures, decreased responsiveness, dyskinesias, and other movement abnormalities and/or autonomic instability are frequently seen as well. We present the case of a woman who was followed up at our facility for over 14 years for the treatment of multiple neuropsychiatric symptoms. Initially, she presented with paresthesias, memory loss, and manic symptoms. Nine years later, she presented to our facility again, this time with left sided numbness, left eyelid droop, and word finding difficulties. Finally, five years later, she presented with manic symptoms, hallucinations, and memory impairment. During her hospitalization, she subsequently developed catatonic symptoms and seizures. During her stay, it was discovered that she was positive for anti-NMDA receptor antibodies and her symptoms responded well to appropriate therapy. This case demonstrates that it may be useful for clinicians to consider screening for anti-NMDA receptor antibodies in long-term patients with neuropsychiatric symptoms that have not adequately responded to therapy.

## 1. Introduction

Anti-N-methyl-D-aspartate (NMDA) receptor encephalitis is an autoimmune disorder characterized by IgG autoantibodies directed against the NR1 subunit of the NMDA glutamate receptor. The disease commonly occurs in young females and frequently is associated with ovarian teratoma, but cases have been reported in males and females of all ages (8 months to 85 years), with or without teratoma [1, 2]. The majority of patients experience prodromal symptoms, including headache, fever, nausea, vomiting, diarrhea, or upper respiratory-tract symptoms. Within a few days, and typically fewer than two weeks, patients exhibit psychiatric and cognitive abnormalities which progress to seizures in the initial stage of the disorder. This is often followed days to weeks later by decreased responsiveness that may alternate between periods of agitation and catatonia, associated with neurological findings including dyskinesias (especially orofacial) and other abnormal movements (e.g., limb and trunk choreoathetosis, elaborate motions of the arms and legs, oculogyric crisis, and spastic rigidity). Patients simultaneously develop autonomic instability, characterized most frequently by hyperthermia, tachycardia, hypersalivation, hypertension, bradycardia, hypotension, urinary incontinence, and erectile dysfunction. Cardiac dysrhythmias and hypoventilation may ensue, necessitating pacemaker placement or intubation and mechanical ventilation [1, 3].

In the initial phase of the disorder, patients may present with a number of psychiatric findings, including anxiety, insomnia, confusion, psychosis (delusions and/or auditory or visual hallucinations), mania, depressed mood, aggression, short-term memory loss, emotional disturbances, and speech abnormalities (e.g., reduced verbal output, frank mutism, and echolalia) [1, 2, 4]. While these signs and symptoms may be accompanied by the florid neurological deterioration described above, milder or incomplete forms of anti-NMDA receptor encephalitis have been observed in a subset of patients (4% in a recent cohort of 571 patients with IgG antibodies against the NR1 subunit of the NMDA receptor), with apparently isolated psychiatric symptoms, seizures, or dystonia [1, 4]. These patients do not necessarily progress to severer disease despite prolonged periods without treatment [4].

In this paper, we present the case of a patient who experienced a severe, more typical manifestation of anti-NMDA receptor encephalitis following a history of previous psychosis and neurological abnormalities.

## 2. Case Presentation

The patient was a mixed-race female (father Caucasian, mother East Asian) who presented to our facility at the age of 24 with disorganized thinking, increased energy, increased appetite, increased libido, labile mood, and abnormal sleep patterns. In the six months prior to this episode, she had visited the outpatient neurology clinic on several occasions for bilateral upper extremity paresthesias and weakness. During the initial interview at our facility, the patient displayed marked word-finding difficulty and short-term memory impairment. At the time, she was not receiving treatment with any psychotropic medication. Head CT and MRI of the brain were normal. EEG was interpreted as showing mild, diffuse slowing. The patient was treated with olanzapine 10 mg PO QD and valproic acid 250 mg PO TID with improvement in manic symptoms, although two weeks later at time of discharge she still appeared confused, inappropriately answering multiple questions by stating, “I feel fine, I feel fine.” She was discharged to a psychosocial rehabilitation and recovery center (PRRC) with a diagnosis of schizoaffective disorder, bipolar type.

Ten days later, the patient returned to our facility with increased agitation, disorientation, impaired attention, auditory hallucinations of singing voices, left-sided paresthesias, and a delusional belief that she was pregnant. Since being discharged to the PRRC, the patient had become unable to answer simple questions or follow simple directions. She was admitted to our facility and stayed as an inpatient for 30 days, during which time she was given trials of several neuroleptics—including haloperidol 2 mg PO BID and mesoridazine 50 mg PO BID—with minimal improvement in her disorganized thinking. When she was discharged again to the PRRC, the patient continued to display impaired attention and was unable to perform serial 3s or spell “world” backwards. During her stay at the rehab center, the patient exhibited persistently disinhibited and hypersexual behavior for several months. She had to be prevented from compulsively overeating and was noted to sneak away to eat large amounts of ice cream out of a bucket. The patient did recover gradually, however, and after a 4-month stay at the rehab center had returned to her preillness baseline mental status. She then maintained regular follow-up appointments in our outpatient psychiatric clinic for supportive therapy and medication management.

Nine years after the previously described hospitalization, at the age of 33, the patient presented to our outpatient psychiatric clinic with speech problems. For 2 weeks, she had experienced word-finding difficulty, stating, “It's like my brain is not connected to my mouth.” In addition, she reported intermittent left-sided eyelid drooping and left-face and left-arm numbness, while denying any weakness. Her only psychotropic medication at the time was lamotrigine (maximum dose 200 mg PO QD) for mood stabilization. On physical exam, she demonstrated left-sided ptosis with continuous upward gaze; otherwise, her neurological exam was without abnormalities. Brain MRI was normal. Serology to evaluate for myasthenia gravis was negative, and EMG/NCS with repetitive stimulation was normal. Our neurology service was consulted; given the lack of specific findings suggestive of myasthenia gravis or cerebrovascular accident, the patient was managed conservatively. At a follow-up visit five months later, her previous signs and symptoms had resolved spontaneously. The patient then continued to maintain regular follow-up with our outpatient psychiatry clinic.

At the age of 38, the patient presented to our emergency department with labile mood, confusion, agitation, easy distractibility, and insomnia. For the previous one month, she had experienced short-term memory impairment and frequent auditory hallucinations of electronic dance music, for which our outpatient psychiatry clinic had placed her on aripiprazole 5 mg PO QD as well as increasing her dose of lamotrigine from 200 mg to 250 mg PO QD, although the memory problems and hallucinations had persisted. Her only psychotropic medications at this time were lamotrigine and aripiprazole (doses as above), and she reported strict compliance. While being interviewed in the ED, the patient exhibited both thought blocking and frequent word-finding difficulty.

The patient was admitted to the inpatient psychiatric ward with a diagnosis of “bipolar I disorder, mixed with psychotic features” and began to manifest catatonic symptoms, including mutism, waxy flexibility, and posturing, alternating with undirected motor agitation and yelling. Six weeks after admission, she developed autonomic instability with fever to 102°F and CPK > 18,000. Aripiprazole was discontinued due to concern for neuroleptic malignant syndrome; despite the absence of rigidity, dystonias, or leukocytosis, it was determined that rhabdomyolysis in the setting of catatonia was a more likely diagnosis. The patient's lamotrigine was also discontinued at this time, and once her CPK level had returned to normal, she underwent three ECT treatments for her refractory catatonia. Prior to the fourth scheduled treatment, she experienced several generalized tonic-clonic seizures and was transferred to the ICU and intubated. Video EEG confirmed the diagnosis of generalized tonic-clonic seizures but was limited by significant muscle and movement artifacts. However, based on the clinical semiology and subtle electrographic features prior to generalization, the seizures likely originated in the left frontal lobe. MRI brain showed no abnormalities. Anti-NMDA receptor antibodies were detected on serological testing, confirming a diagnosis of anti-NMDA receptor encephalitis. Oncologic workup with CT chest, CT abdomen/pelvis, and PET/CT from skull base to mid-thigh was negative for any tumor or malignancy. The patient received high dose IV corticosteroids (methylprednisolone 1 g/day) and IVIG (Gamunex 0.4 g/kg/day) for five days with resolution of catatonia, although her cognition remained impaired. She underwent a second course of high dose IV corticosteroids (methylprednisolone 1 g/day) for 7 days with improvement in mental status, although she continued to display memory impairment, slurred speech, and impaired attention. The patient was discharged on prednisone 60 mg PO QD (1 mg/kg) and continued treatment as an outpatient. Her psychotropic medications at discharge were clonazepam 2 mg PO QID for anxiety and valproic acid 500 mg PO BID for mood stabilization and seizure prophylaxis. Since that time, the patient gradually has been weaned off of prednisone over the course of one year. She experienced improvement in cognition over several months, and at a follow-up visit in our clinic eight months after discharge she was clearly at her premorbid level of cognitive function. At her most recent outpatient clinic visit, one year after discharge, the patient continued to be at her baseline level of cognitive function with no return of psychiatric or neurologic symptoms. At this time, her only psychotropic medication is lamotrigine 100 mg PO QD for mood stabilization.

## 3. Discussion

A recent cohort study of 571 patients with IgG antibodies targeting the NR1 subunit of the NMDA receptor found that 23 (4%) of these patients developed isolated psychiatric episodes (defined by the authors as lacking neurological involvement), either at disease onset or relapse [4]. Some of these episodes might be characterized more accurately as limited forms of the disease rather than isolated psychiatric episodes, as mild neurological abnormalities (e.g., orofacial dyskinesias, memory problems, and hypersalivation) were found on exam, along with abnormal but nonspecific findings on EEG (either epileptic or nonspecific slowing) and MRI (including nonspecific fluid-attenuated inversion recovery changes involving temporal, frontal, and/or parietal lobes; mild atrophy of temporal lobes; and diffusion-restricted abnormality in the corpus callosum). Moreover, analysis of the 571 patients in this study showed that those with monosymptomatic or milder presentations do not necessarily progress to more severe multisymptom disease, despite prolonged periods without treatment. The fact that the five patients in this study who presented with limited forms of the disorder at disease onset were tested for anti-NMDA receptor antibodies only after abnormal findings were discovered on MRI suggests that this condition might be underdiagnosed in patients without abnormal findings on ancillary testing [4]. Moreover, the identification of this limited form of anti-NMDA receptor encephalitis begs the question of what the natural history of the disease would be if these patients are not treated with immunotherapy and, when applicable, tumor removal.

In the present case, our patient initially presented to our facility at the age of 24 with psychosis, confusion, labile mood, cognitive impairment, and paresthesias, with diffuse slowing noted on EEG. During her recovery from this first episode, she displayed persistent impairment in executive function with impulsivity and disinhibition that resolved over the course of several months. In retrospect, we propose that given the subsequent detection of anti-NMDA receptor antibodies during her presentation at the age of 38, her earlier episodes may have represented limited presentations of anti-NMDA receptor encephalitis. This possibility appears more likely given that an EEG performed at the age of 28 for an isolated syncopal episode (the patient was otherwise psychiatrically and neurologically asymptomatic at this time) showed no abnormal slowing or other abnormal findings. Several recent studies have shown that patients with a history of psychosis associated with bipolar disorder, schizophrenia, or schizoaffective disorder tend to display persistent EEG abnormalities even when not actively psychotic [5, 6]. It appears that EEG abnormalities in anti-NMDA receptor encephalitis, on the other hand, tend to resolve with clinical improvement [3, 7].

The obvious limitation of our hypothesis is that anti-NMDA receptor antibodies were not tested, and therefore not detected, in the patient until she presented with florid disease at the age of 38. However, given the aggregate of less specific findings (psychosis, confusion, memory impairment, dyskinesias, speech and language problems, impaired executive function, and EEG abnormalities) from previous episodes that collectively resist explanation by an alternative diagnosis, we believe it is reasonable to suggest anti-NMDA receptor encephalitis as a retrospective diagnosis that would account for these findings.

This case is significant because, to our knowledge, if our patient's previous episodes are in fact attributable to pathogenic anti-NMDA receptor antibodies, this would represent the most extensive illustration to date of the natural history of the disease in a patient not initially treated with immunotherapy or tumor removal, characterized by relapsing and remitting symptoms subsequently progressing to florid disease with seizures, dyskinesias, and autonomic dysfunction, ultimately requiring intubation and mechanical ventilation. Previous case series have described patients who spontaneously recovered from the disease without receiving immunotherapy or tumor removal and also patients who have experienced multiple relapses of the disease after treatment [3, 8].

In terms of practical implications, we believe our patient's case supports the recommendations of others to test for anti-NMDA receptor antibodies in cases of psychosis (both first episode and recurrent) with neurological abnormalities (e.g., dyskinesias, speech and language problems) and/or abnormal findings on ancillary testing (EEG, MRI) [4]. When our patient first presented to our facility at the age of 24 with psychosis and neurological symptoms, anti-NMDA receptor encephalitis had not yet been characterized as a clinical entity. However, given that anti-NMDA receptor antibodies have been found to remain detectable after recovery from the acute illness, it is not unreasonable to suppose that our patient might have tested positive for these antibodies had serology been performed sometime between this initial hospitalization and her final presentation at the age of 38 with florid disease [9].

As early detection of antibodies may allow for earlier treatment of anti-NMDA receptor encephalitis, which is associated with better outcomes, we believe the present case underscores the importance of clinicians maintaining vigilance for neurological abnormalities, which may be subtle, in psychotic patients, and performing serological testing according to clinical judgment [3, 10, 11]. Whether the threshold for antibody testing should be lowered further to include all patients presenting with first episode psychosis, as some have recommended, will depend on the findings of future prospective cohort studies that better assess the prevalence of anti-NMDA receptor antibodies in this patient population [4, 12].
